# Sponge Gold

**Published:** 1854-07

**Authors:** W. H. Dwinelle


*Sponge Gold.* By W. H. DWINELLE, M. D., D. D. S.

To produce a gold stopping of the highest excellence with foil, two things at least are absolutely necessary. First, the possession of a material that shall combine the qualities of toughness, adhesiveness and purity. Second, the best instruments in the hands of the most skillful experience.

But however excellent the appliances, well adapted the instruments, or ingenious the hand that guides them, they are all valueless, and fail to accomplish the end, if the gold does not combine the qualities above indicated.

To insure with uniformity the characteristics of adhesiveness and toughness, it is absolutely essential that the gold should first be made *pure.*

For the present, it is sufficient to remark, that much of the gold prepared for our profession is rendered "chemically pure" by *quartation,* and consequently can not always be pure, for the reason that gold often contains foreign metals not soluble in acids. In addition to this, gold is often alloyed before it is beaten into foil.

Indicative of this fact, and as a universal test of the quality of gold, whether in the form of foil or otherwise, it is only necessary to consider that *pure gold is of a uniform color throughout the world.* A comparison of Abbey's foil, and some few others with the paler foils of the market, will render it easy to distinguish between gold which is absolutely pure, and that which is not.

At some future time we will endeavor to give in the Journal the process by which gold may be obtained, in *all cases,* entirely pure. But to our purpose.

Although we must concede that the best operators in our profession are enabled, with a superior article of foil, to produce stoppings of a degree of perfection very nearly approaching all that is desirable in our art, yet it must be admitted that the *ultimatum* is not yet reached; a *perfectly solid* stopping has not yet been made with foil—a perfectly solid mass whose particles are so entirely integrated, that it may be rolled out into a plate or drawn into a wire, and possess all of the characteristics of plate and wire obtained from melted gold. To be met here with the statement that gold stoppings are already produced of such a high degree of excellence as to answer all practical purposes, or even to endure every test but the last referred to, does not reflect upon what we have assumed, or divert us for a moment from our object. We may be critical, but we are aiming at perfection.

If the experience of the future shall prove that an article of plastic gold can be produced, so conditioned that its particles may be consolidated into a mass, so perfect as to answer all of the tests that could be applied to melted gold, we are bound to regard it as a higher approximation to perfection than any hitherto attained.

The needs of the profession in this direction have long been felt and acknowledged; and it has been the constant aim of those most anxious for the advancement of our art, to produce an article which would endure these high tests.

To this end, various attempts have been made to produce an article of *sponge gold*, or gold in such a finely divided condition, that on pressure it will weld into a solid mass.

In the July No. of the Journal, reference is made to the process by which several kinds of sponge gold have been produced. We will allude to these.

First, that produced by oxalic acids. Although it is precipitated in a minute state of division, it is exceedingly brittle, partakes of the quality of a powder rather than a porous mass, is difficult to introduce into the cavity of a tooth, and when introduced, often gives a brown stain to its surrounding walls.

Sponge gold, made by fusing gold and silver together, and then extracting the latter by nitric acid, though better than the article just referred to, is of poor quality, in consequence of the imperfect manner in which the silver is extracted, from its being in comparative large masses, and also in consequence of the presence of foreign metals not soluble in acids. It lacks the quality of toughness, is managed with great difficulty, and is subject to great waste.

That produced by *aqua regia*, though liable to be impure, is still far superior to either of the others, and until recently represented the highest advancement in this direction; though friable, it is more plastic and manageable, and with sufficient time and proper care, can be resolved into a stopping of great solidity.

While in Europe, a little more than a year ago, Mr. John Tomes, surgeon dentist to Middlesex Hospital, London, showed us an article of sponge gold, uniting more desirable qualities than we had hitherto any knowledge of. It occurred in irregular rounded masses or pellets, a little larger than an ordinary sized pea. Its surface was of lighter hue than its appearance within, glistening at different points, as though, since its formation, it had been subject to a high degree of heat, yet without diminishing it softness or pliability. On breaking it open its peculiar spongy character manifested itself in a most beautiful degree, its infinitesimal particles uniting together, forming a dense and delicate network. We filled two or three extracted teeth with it, forming large and exceedingly hard stoppings. After polishing these, with a graver we engraved lines and letters upon them, which, on being subjected to a powerful lens, displayed the angles of each groove as clean and sharp as though it had been cut upon jewelry.

The only objection that could be urged against the gold referred to, was its want of a sufficient degree of plasticity, its disposition to crumble on being broken, and consequent liability to waste in using. It seems, however, that Mr. Barling, the maker of the article, has recently succeeded in overcoming this objection to a great degree.

To Mr. Joseph Barling, No. 9 High street, Maidstone, Kent, is the profession indebted for this beautiful article, so full of promise to our profession.

At the same time, and without any knowledge of the experiments or success of Mr. Barling, Dr. A. J. Watts, Chemist, of Utica, N. Y., was pursuing a series of chemical experiments with reference to obtaining an article of sponge gold which should supply the wants of our profession, and shortly after our return, he placed in our hands for trial three different articles of sponge or minutely divided gold.

The first is of a highly crystalline character. The second is in lamina, made up of exceedingly fine granules. The third is in a spongy aborescent form.

The first, or crystalline gold, with proper care and handling, forms a solid plug; but unless great care is used, is subject to considerable waste.

The second, or laminated gold, is a much better article from its tougher character and extreme adhesiveness, but from the thinness of its plates, the operation of filling is rendered extremely slow.

The third, or sponge gold proper, is in the form of a cake, from one-eighth to one-fourth of an inch in thickness, of a compact, spongy, aborescent character; possessing, in the most eminent degree, all of the desirable qualities of the above—toughness, compactness, pliability, together with plasticity, and the highest degree of adhesiveness.

The method of producing the first, or crystalline gold, is familiar to the readers of the Journal. The mode of preparing the second and third is not yet published, but they are evidently prepared in an entirely different manner from the first.

With this last article we have had considerable experience, and with uniform satisfaction, especially in large stoppings.

In using the sponge gold, we adopt the following method: With a sharp blade we cut off from the cake of gold a sufficient quantity for our present purpose; this we anneal thoroughly with an alcohol lamp, and then, spreading it upon a clean paper before us, we cut it up into fragments and pellets best adapted to the cavity into which it is to be introduced.

Being previously provided with various instruments, whose extremities are subdivided into two or more points, we, by pressure upon the sponge, readily induce it to adhere to them, when we carefully carry it to its destination in the cavity of the tooth, which has been previously dried with paper. As the operation is repeated, accompanied with thorough packing and pressure, it will be found that the particles of gold readily weld together into a solid mass; so that when the stopping is completed, it in all respects resembles melted gold, and may be subjected to the same treatment with impunity. For the purpose of determining its various qualities as a stopping for the teeth, we subjected it to the following tests:

To test its *malleability*, we took a large plug of gold formed in the manner just described, laid it upon an anvil, and with a hammer beat it to flatness; annealing it, we passed it through a rolling mill, when it was formed into plate, as perfect in all its characteristics as any plate made of pure gold.

To test its *ductility*, we took a similar plug, formed as before, and drew it out into wire as fine as No. 80, Stubbs' plate.

To test its corking or stopping quality, and the impermeability of its antagonizing *joints*, to fluids, we took a piece of thick glass tube, about a foot long, into one end of this, to the depth of more than half an inch, we introduced a stopping of sponge gold. Inverting the tube, we poured into it a solution of red saunders; we then closely fitted a piston and rod to the tube immediately above the fluid, and upon this applied a weight. At the expiration of twenty-four hours, the fluid had not made the slightest progress downwards.

To test its ability to being built up into irregular and independent shapes, we have repeatedly reproduced from one-half to three-fourths of the entire crowns of molar teeth, *in gold.* As a farther test, we took a block of ivory, *chucked* it upon our lathe, and with small tools, formed a matrix to correspond to the size of a large finger ring. Into this we introduced, by packing and condensing, as in stopping teeth, more than five dwts. of sponge gold; placing it back upon our lathe, we turned out the ivory within and without the golden circle, until it became entirely separated; this readily endured all of the necessary process of filing, stoning and burnishing, into a beautiful massive gold ring, which has been worn constantly for several months, and will, in all respects, stand trial with any pure gold ring made in the ordinary way. It has this advantage, however, over all rings made heretofore, it *is* a *ring*, an uninterrupted ring, and "has no end," a continuous circle, with no *alloy* between!

As a test of density, well formed plugs do not shrink under the blow-pipe; their inner surfaces are bright and solid, while their polished disks take the graver like plate.

Under the microscope it presents a beautiful and gorgeous appearance, like looking into a golden sylvan grove, each mossy or aborescent branch being in the form of a six sided crystal.

Although we consider Dr. Watts' sponge gold indispensable to our practice, yet we do not think it will ever entirely supercede the use of gold foil. It can often be used to great advantage in combination with gold foil. In large stoppings, it possesses great advantages over foil, from the facility with which it can be introduced, and consequent freedom from the fatigue which ever accompanies long operations.

We think no one in our profession, who has had experience in its use, would be willing to be without it.

Dr. Watts is deserving of great praise for his persevering course of experiments, which have resulted so favorably to our art. May he reap the abundant reward he deserves.

